# *Acheta domesticus* (Cricket) feed resources among smallholder farmers in Lake Victoria region of Kenya

**DOI:** 10.1002/fsn3.1242

**Published:** 2019-12-09

**Authors:** Jackline A. Oloo, Monica Ayieko, John Maina Nyongesah

**Affiliations:** ^1^ Department of Agricultural Economics and Agribusiness Management Jaramogi Oginga Odinga University of Science and Technology Bondo Kenya; ^2^ School of Agriculture and Food Sciences Jaramogi Oginga Odinga University of Science and Technology Bondo Kenya; ^3^ Department of Biological Sciences Jaramogi Oginga Odinga University of Science and Technology Bondo Kenya

**Keywords:** crickets, feeds, food, nutrition

## Abstract

The quality of domesticated crickets depends on the choice of feed substrate that has a direct impact on the economic viability of the farming operation. This study assessed the popular local cricket feeds in the Lake Victoria region. Data were collected through individual interviews, focus group discussions, personal observations, and growth experiments. Kales, sweet potato leaves, ugali, and banana peels were the most preferred by the farmers. Crickets fed on kales and sweet potatoes had a significantly higher weight gain and maturity rate than those fed on ugali and banana peels (*p* < .05). Additionally, crickets fed on kales and sweet potatoes had higher (82.4% and 78.6%, respectively) crude proteins compared to those fed on ugali (57.9%) and banana peels (62.9%). Kales and sweet potatoes can serve as cheaper, alternative local feeds for cricket farming. Empowerment of farmers through subsidies or provision of loans can enhance local cricket feed production.

## Graphical abstract

Cricket rearing regions in Kenya.

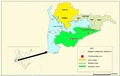

## 1 INTRODUCTION

The considerable attention devoted to upscale cricket production in Kenya echoes the efforts made to promote edible insects to meet food and feed demands globally. Despite these efforts, little attention focuses on examining whether local cricket feed ingredients used by farmers are present in sufficient quantities and have nutrient densities in recommended levels for optimal cricket growth performance. The quality of domesticated insects depends heavily on the quality, consistency, and choice of feed substrate, which has a direct impact on the economic viability of the farming operation (Miech et al., 2016; Van Huis et al., 2013; Vantomme, 2015). An insect's nutrient requirements will change with time because of the varying demands for growth, reproduction, or diapause (Chapman, Simpson, & Douglas, 2013; Lundy & Parrella, 2015). Seasonal variability of feed also has an effect on the behavioral and physiological adaptations of wild insects (Dunbar & Gassmann, 2013; Mabry, Spencer, Levine, & Isard, 2004; Sisodia & Singh, 2012; Van Huis et al., 2013). The abundant nutrient in the diet will be expressed as a higher percentage of the cricket's dry mass than produced by a normal diet (Chapman et al., 2013). A balanced diet is therefore crucial for the optimal growth of domesticated crickets since it will determine their maturity rate, reproduction performance, and resultant nutrient composition (Arganda et al., 2014; Oonincx & van der Poel, 2011; Raubenheimer & Simpson, 2003; Yaday, 2003).

Cricket production in Kenya, like in many parts of the world, is dependent on a variety of feed resources mainly including vegetables and commercial feed (Ayieko, Ogola, & Ayieko, 2016; Halloran, Roos, Flore, & Hanboonsong, 2016). The quality and quantity of cricket feed may also have an impact on the current price of a kilogram of crickets sold by farmers in Kenya. Local feedstuffs can be sustainable for optimizing production in general due to their availability and high nutrient content. For instance, amaranth is associated with high nutritional value, high yields, and ability to grow in hot climates, thus are popular as vegetables as well as a fodder crop in most parts of Kenya (Kamotho, Kyallo, & Sila, 2017; Ndungu, Kuria, Gikonyo, & Mbithe, 2017; Njoroge, Onyango, Mugera, Kinyuru, & Mwaniki, 2017). Similarly, Moringa leaves are rich in nutrients as fresh leaves or cooked and are in full leaf at the end of the dry season when other foods are typically scarce (Capper, Berger, & Brashears, 2013; Fahey, 2005; Hanboonsong & Durst, 2014). Cambodian field crickets have also been shown to perform well on cassava plant tops and *C. rutidosperma* (Miech et al., 2016). A comparative study on crickets fed on agro‐byproducts (Orinda, Mosi, Ayieko, & Amimo, 2017) revealed the potential use of agro‐byproducts as a cheap source of cricket feed.

In Kenya, farmers earn an average of USD 6.77 per kilogram of freshly harvested crickets (Halloran et al., 2016). This price is higher than the price per kilogram of other popular proteins such as beef, mutton, chicken, fish, and legumes (FAO/GOK, 2018). This implies that farmers have an opportunity to skim the market through the sale of crickets. Additionally, the use of local feedstuffs could decrease the cost of feed due to their adaptability to local geographical conditions, nutrient value, and high probability of widespread use due to their use as human food.

Insufficient documentation of a geographical and nutrient profile of locally available feeds that have positive nutrient and growth‐enhancing aspects for domesticated crickets weighs down efforts to promote insects as food and feed. The high cost of cricket feed continues to be the main challenge in countries with established cricket farms (Hanboonsong & Durst, 2014; Van Huis et al., 2013). A geographical profile of local feed resources would greatly inform local farmers on the available feeds within their region and increase the efficiency of cricket production, especially among smallholder farmers. This study assessed the growth performance of crickets fed on popular feed resources in the cricket growing regions of the Lake Victoria region of Kenya.

## 2 METHODOLOGY

### 2.1 Study areas

**Figure 1:**
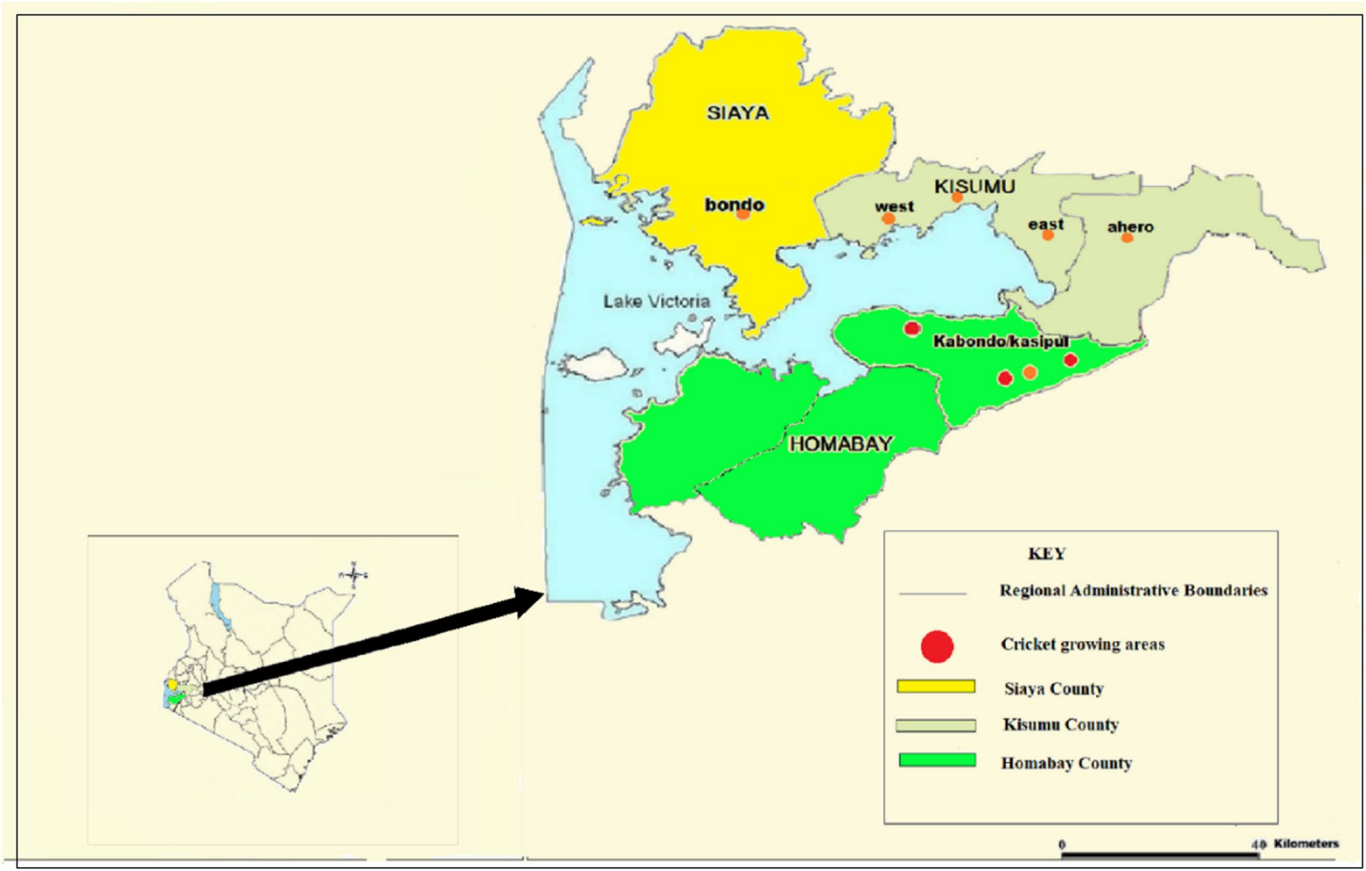
Map of Kenya with the study regions highlighted in yellow (Bondo Subcounty in Siaya), jungle green (Kisumu East and Kisumu West Subcounty in Kisumu County), and bright green (Kabondo/Kasipul in Homabay County)

This study was carried out in Bondo, Kabondo, and Kisumu West and Kisumu East Subcounties (Figure 1) located within the Lake Victoria region of western Kenya during the period of October 2015 to August 2016.

Bondo Subcounty is situated in Siaya County in Nyanza Province along Lake Victoria. It lies between 0°26° and 0°90° from longitude 33°58°E and 34°35°W and covers a total of 1,972 km^2^ out of which 972 km^2^ is landmass while the rest 1,000 km^2^ is water surface. Its population is projected to be 275,543 (DEAP, 2007). Out of the 972 km^2^ total land area, 796 km^2^ is arable, although only about 40% of this area is currently under arable farming. The farms are average in size (~3 ha) with mainly subsistence mixed crop and livestock farming being carried out that contribute to 79% of the household incomes. Extremely low yields and low input level characterize the farming system in this region (Achonga, Lagat, & Akuja, 2011). The predominant soils are mainly Cambisols and Vertisols with low‐to‐moderate fertility (FAO, 1997). Rainfall is low and ranging between 800 and 1,600 mm on bimodal rainfall pattern of long rains occurring between March and May and short rains occurring between October and November.

Bondo has a tropical climate with an average temperature of 22.5ºC, and evaporation varies between 2,000 and 2,200 mm annually (DEAP, 2007). Main food crops grown include sweet potato, maize, cassava, groundnuts, beans, and sesame.

Kabondo Kasipul Subcounty is situated in Homabay County of Nyanza Province. It lies between latitude 0^o^15′ South and 0°52′ South, and between longitudes 34° East and 35° East and covers a total area of 509.5 km^2^. Its population is projected to be 179,788 (KNBS, 2010). It is characterized by small farm sizes averaging 2 ha per household. The predominant soils are nitosols, cambisols, and regosols, which are relatively fertile and well‐drained. Coupled with a modified inland equatorial climate, which is influenced by altitude and its nearness to Lake Victoria, makes the subcounty rich in agricultural productivity. It receives an annual precipitation of 1,100‐1,600 mm per annum. There are two rainy seasons, namely the long rainy season from March to June and the short rainy season from August to November. Temperature ranges from 14 to 28°C. Main food crops grown include coffee, tea, pineapples, sorghum, sweet potatoes, millet, fruits, green grams, vegetables, groundnuts, sugarcane, bananas, sorghum, maize, groundnuts, and beans. This study interviewed farmers from Nyapalo, Kadongo. Administratively, Kabondo Kasipul Subcounty is divided into 23 locations and 58 sublocations. This study interviewed farmers who were purposively selected from Kadongo, Kotutu, Oriang, and Nyapalo in the western part of the subcounty.

Kisumu County lies within longitudes 33°20′E and 35°20′E and latitudes 0°20′South and 0°50′South. The county covers a total of 2,576.5 km^2^ out of which 2,009.5 km^2^ is land area and the remaining 567 km^2^ is covered by water. It has a total population of approximately 968,909 people (KNBS, 2010). The county has seven subcounties, namely Kisumu West, Kisumu Central, Kisumu East, Seme, Muhoroni, Nyando, and Nyakach. This study interviewed cricket farmers in Kisumu West and Kisumu East subcounties. Kisumu West has 33,232 households and covers a land area of approximately 212.90 km^2^ with a population density of approximately 131,246 people (KNBS, 2010). Cricket farmers interviewed were located in Osiri and Ojolla sublocations within Kisumu West. Kisumu East has a population of approximately 150.124 people and covers approximately 135.90 km^2^. This study interviewed cricket farmers located in Wathorego, Manyatta B, and Migosi within Kisumu East Subcounty. Overall, the main food crops grown in Kisumu County include maize, cassava, beans, groundnuts and sweet potatoes; while the main cash crops are tea and coffee.

#### 2.1.1 Field survey on the types and availability of local feeds for domesticated crickets

A structured questionnaire was administered to 43 purposively sampled farmers from Bondo, Kabondo, and Kisumu East and Kisumu West Subcounties and included 10, 11, 9, and 12 farmers, respectively. Farmers were asked questions on cricket feed types and availability.

#### 2.1.2 Effect of local feeds on the growth performance of domesticated crickets

Experiments were conducted to find out the effect of local feeds on the development of domesticated crickets.

### 2.2 Cricket rearing

Experiments were conducted using 14‐day‐old nymphs of *Acheta domesticus* species that were hatched at the Jaramogi Oginga Odinga University demonstration farm prior to the experiments. The use of live nymphs made it easier to estimate the number required for each feed type and to follow up on growth parameters, since the eggs’ microscoping membranes could easily break if counted. The nymphs were hatched on 100‐liter buckets and fed on growers mash for the first 14 days after hatching to acclimatize to the prevailing experimental conditions and allow all cricket populations to attain a uniform metabolic capacity prior to the start of the feeding experiment. Fifty randomly selected nymphs were then counted and transferred to each experimental unit and kept for five days to allow adaptation to experimental conditions.

### 2.3 Experimental feed types

Field data on popular local cricket feed were analyzed. The results demonstrated that kales (*Brassica oleracea var. ocephala*), banana peels (*musa acuminate*), sweet potato vines (*Ipomoea batatas*), and ugali were most commonly used as cricket feed across the study regions. Thus, a follow‐up study was conducted to determine the effects of these feed resources on growth parameters of crickets. Growers mash was used as a positive control feed. Feed was offered ad libitum for a 14‐week period. Sweet potato leaves were freshly picked from the garden while kales were purchased every day and cleaned by washing before being fed to crickets. Ten kilograms of growers mash purchased from “Unga feeds” Company was ground using an electric mill to break down into flour and avoid wastage. *Ugali* is corn cake prepared by stirring cornflour in boiling water. The cornflour is added into the boiling water while stirring to achieve the softness that is the characteristic of ugali. Ripe bananas were purchased daily and washed before peelings were offered to the crickets. This initial treatment of feeds helped to eliminate disease‐causing micro‐organisms.

### 2.4 Experimental design

Five experimental feed treatments derived from the popular local feed resources were used in this study, namely (a) growers mash as a positive control, (b) kales, (c) banana peels, (d) sweet potato vines, and (e) *ugali*. Each of the five feed types was provided to 50 cricket nymphs and replicated thrice resulting in a total of 15 experimental units (Table 1). Reports by Hanboonsong et al. (2013) indicate that cylindrical cages that were 80 centimeters in diameter and 50 centimeters high can produce around 2–4 kg of crickets. This implies that the current study offered good congestion‐free environment for the nymphs to grow. The population of 50 crickets per experimental unit also reflected the number of uniformly hatched crickets, that is, those that hatched at the same time and that were readily available at the JOOUST demonstration farm where the nymphs were sourced.

**Table 1:** Experimental design showing the type of experimental feed, the number of crickets used per replicate, and the total number of crickets per treatment

Type of experimental feed/treatment	Number of crickets/replicate	Number of replicates	Total number of crickets
Growers mash	50	3	150
Sweet potato vines	50	3	150
Ugali	50	3	150
Kales	50	3	150
Banana peels	50	3	150

The feeds were provided to nymphs 14 days after hatching. This is because absolute biomass gain is very small during the early developmental period, yet growth rates in the early developmental stages can affect growth rates throughout the life cycle (Lundy & Parella, 2015). Therefore, a balanced uniform feed (i.e., grower mash) provided to all nymphs during the first 14‐day period ensured that all cricket populations have a uniform metabolic capacity prior to the start of feeding experiment (Lundy & Parella, 2015). This enhanced the precision of determining the effect of feed type/treatment on growth parameters of crickets. All the nymphs used were of the same age as all of the eggs hatched uniformly at the same time. Each experimental feed was provided ad libitum. At the start of each day, old feed provided the previous day was discarded and fresh feed was offered per experimental unit. Feed was provided ad libitum to the crickets to consume until they were fed. This ensured that cases of attrition were not as a result of feed rationing. The nymph crickets were weighed at the start and end of the experiment to determine total weight gain based on the difference between initial weight and weight attained at maturity. After every three days, 25 randomly collected crickets were weighed from each treatment/feed type. Experiments using different feed types were carried out for a 14‐week period to determine their effects on the following growth parameters of crickets, namely (a) weight gain, (b) protein content, and (c) maturation period to adult stage. Thereafter, the mature/adult crickets were harvested by freezing, and proximate analysis was done at the Kenya Bureau of Standards to determine the protein content of crickets fed with the five types of feeds.

### 2.5 Calculation of growth rate

The following formula was used to calculate growth rate:

(1) $$\text{Growth}\;\text{rate}\;=\;\frac{\text{Final}\;\text{weight}\;\text{(gms)}\;\text{(after14}\;\text{weeks)}-\text{Initial}\;\text{weight}\;\text{(at}\;\text{the}\;\text{start}\;\text{of}\;\text{experiment)}}{\text{Maturity}\;\text{period}\;\text{(period}\;\text{at}\;\text{which}\;\text{50\textbackslash{}\%}\;\;\text{attain}\;\text{maturity)}}\;$$

Weight gain was determined by calculating the difference in weight after every three days by using the following formula:

(2) Weightgain=Currentweight-Previousweight(weightbeforethethirdday)

### 2.6 Data collection

A structured questionnaire was prepared and pretested for its applicability before its administration. The questionnaires were administered by the researcher together with trained enumerators. The field officer helped to introduce the selected farmers to the pending exercise. The interviews were conducted on the farm to enable counterchecking of the feed resources available at the farms. Information on cricket feed types was collected using the structured questionnaire. Data on cricket weight gain were recorded at the start of the experiment, after every three days, and at the end of the experiments and entered into excel spreadsheets. At the end of 14 days, crickets were taken to the Kenya Bureau of Standards laboratory for proximate analysis to determine their protein content.

### 2.7 Data analysis

Data from open‐ended questions on availability and types of locally available cricket feed resources were first analyzed to identify similar themes and then coded and entered into statistical package for social sciences (SPSS) for analysis. A *t*‐test with a post‐hoc test was conducted to establish differences in weight gain and to compare the effect of the experimental feeds on the weight gained by crickets during the 14‐week experimental period. Distribution of data was tested for normalcy using residual plots. The level of statistical significance was set to *p* < .05. Frequency, means, graphs, and standard deviation were used to describe statistical summaries such as level of income and feed types used. The Statistical Package for Social Sciences (SPSS) version 17 was used to process and analyze the collected data.

## 3 RESULTS

### 3.1 Popular cricket feed

Kales, sweet potato vines, ugali leftovers, and banana leaves were the popular cricket feeds used by 83.3%, 38.1%, 11.9%, and 45.2% of the cricket farmers, respectively (Table 2).

**Table 2:** Popular cricket feed represented by the proportion (%) of farmers in Bondo, Kabondo, and Kisumu East and Kisumu West Subcounties

Types of feed	Bondo *n* = 10	Kabondo *n* = 11	Kisumu east *n* = 9	Kisumu west *n* = 12	Overall %
Vegetables	Proportion (%)	Proportion (%)	Proportion (%)	Proportion (%)	
Kales	70	100	100	66.7	83.3
Sweet potato leaves	20	80	33.3	25	38.1
Ugali leftovers	20	–	22.2	8.3	11.9
Banana peels	60	40	22.2	58.3	45.2

### 3.2 Growth performance of crickets fed on the profiled feeds

Feeds used in this study varied in their crude proteins as shown in Table 3 below.

**Table 3:** Crude protein composition of experimental feeds used in the 14‐week study on growth performance of domesticated house crickets (*Acheta domesticus*)

Feed	Crude protein	Reference source
Growers mash	15.89%	Orinda et al. (2017)
Kales	26.87%	Acikgoz (2011)
Sweet potato vines	3.02%–7.38%	Ji, Zhang, Li, and Li (2015), Gupta et al. (2018)
Banana peels	6%–9%	Khawas, Das, and Deka (2017), Okorie, Eleazu, and Nwosu (2015), Mohapatra, Mishra, and Sutar (2010)
Ugali	95.0 ± 16.0%	Carter, Dewey, Lukuyu, Grace, and Lange (2015)

Significant differences (*p* < .05) were found among feed treatments. Results of the weight gained by crickets fed with individual experimental feeds after every five weeks are presented below.

#### 3.2.1 Week 1–5

**Figure 2:**
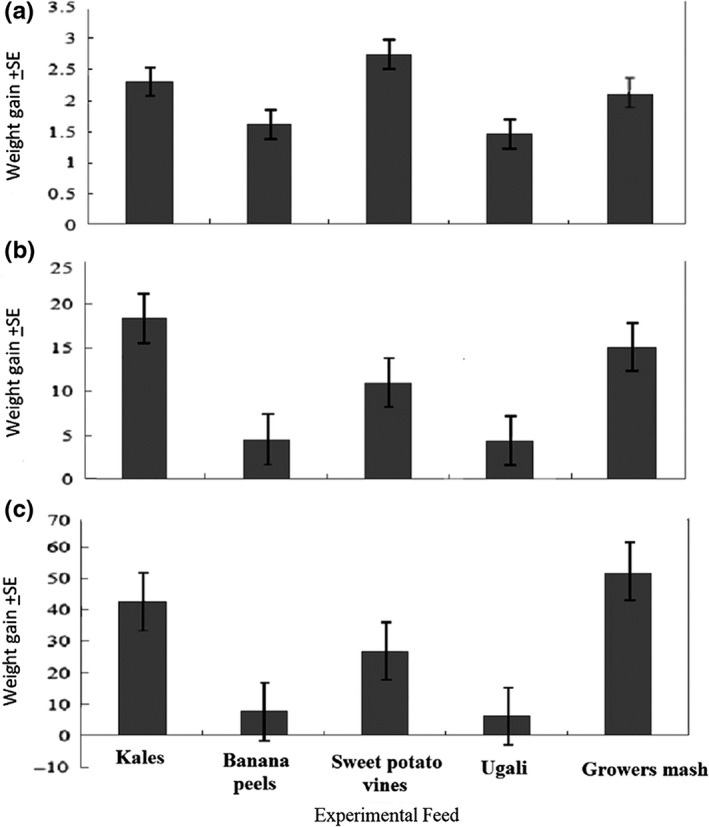
Average weight gain in crickets fed on selected feeds for week 1–5 (a), week 6–10 (b), and week 11–15 (c). Values represent mean ± *SE*

There was no significant difference (*p* > .05, *F*(4, 20) = 0.352, *p* = .83) in weight gained by crickets under different experimental feed treatments. This was evident in the minimal difference in the weight of insects. Crickets fed sweet potato vines had the highest mean weight gain (2.74 + 2.37 g) than the control feed (2.13 ± 1.75 g), followed by crickets fed on kales (2.30 ± 2.28). Crickets fed on banana peels and ugali weighed 1.6 ± 1.2 and 1.4 ± 1.2 g, respectively (Figure 2a).

#### 3.2.2 Week 6–10

The difference in weight between experimental feed groups was statistically significant (*p* < .05), where *p* = .006. Crickets fed on kales showed an increase in weight gain of 18.5 ± 10.06 g, which was 16.16 g higher than their weight in the previous week and 3.3 g higher than the insects fed on control feed (15.15 ± 7.19), while crickets fed on banana peels and ugali showed minimal increase in weight, a trend that continued through the weeks that followed up till the last growth week (Figure 2b).

#### 3.2.3 Week 11–14

There was no statistically significant difference in weight gained by crickets in the week 11 to week 14 of the growth period (*F*(4, 15) = 106.997, *p* > .05). However, crickets that were fed kales responded with a higher final body weight (43.7 ± 4.63 g) as compared to the control feed that recorded the highest weight gain of 52.35 ± 10.89 g (Figure 2c).

**Figure 3:**
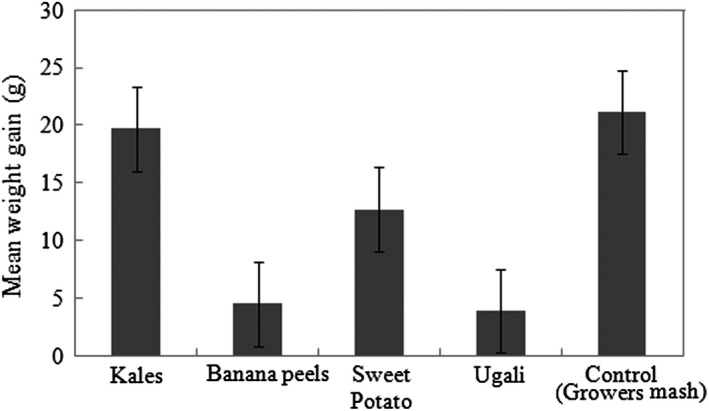
Average weight gain (*g*) of crickets fed on selected feeds for the 14‐week growth period. Value represents mean ± *SE*

Overall, crickets fed on the control feed (i.e., growers mash) showed the highest average weight gain (21.13 ± 5.8 g) at the end of the 14‐week period followed by crickets fed on kales, (19.67 ± 4.7 g). Crickets fed on sweet potato vines had an average weight gain of 12.69 ± 2.9 g. The lowest weight gain was recorded for crickets fed on bananas (4.49 ± 0.75 g) and those fed on ugali (3.93 ± 0.61g) (Figure 3, Table 4). All the experimental feeds used in the growth period produced significantly different results (*p* < .05) (Table 4).

**Table 4:** Statistical comparison of profile feeds used in growth experiment

Sample statistics				Sample test			95% CI	
	Mean	*SD*	*SE*	*t*	*df*	*p* value	Lower	Upper
Kales	19.6786	17.90906	4.78640	4.111	13	.001	9.3382	30.0190
Banana peels	4.4944	2.80877	0.75068	5.987	13	.000	2.8726	6.1161
Sweet Potato	12.6966	10.85942	2.90230	4.375	13	.001	6.4266	18.9667
Ugali	3.9363	2.30901	0.61711	6.379	13	.000	2.6032	5.2695
Control (growers mash)	21.1327	21.95177	5.86686	3.602	13	.003	8.4581	33.8073

**Figure 4:**
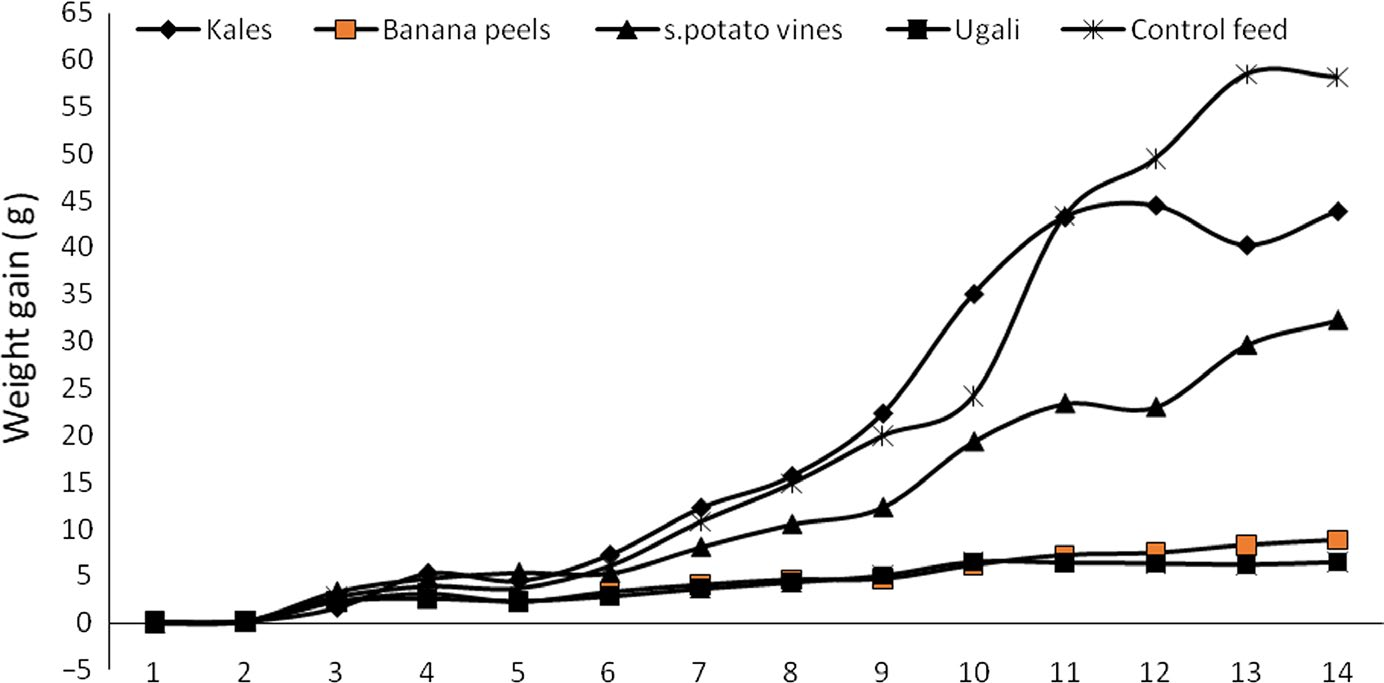
The trend in weight gain during the 14‐week experimental period

Figure 4 shows the general trend in weight gain for the 14‐week period. Both banana peels and ugali exhibited the lowest gain, while the control and kales had the highest weight gain as observed from the trend in weight gain (Figure 4).

### 3.3 Maturation period

**Figure 5:**
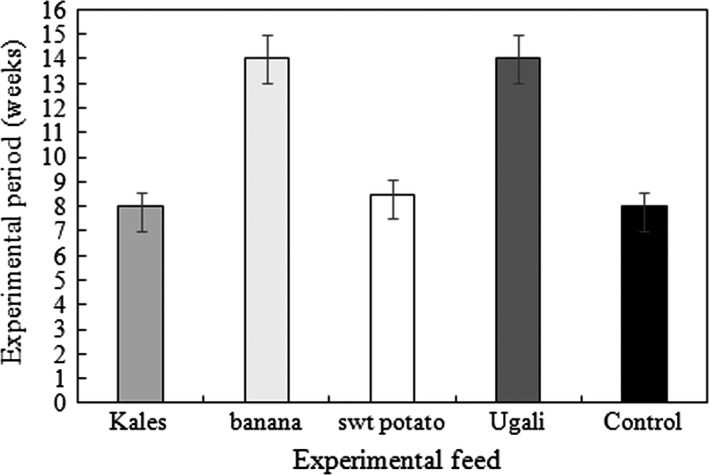
Maturity period of crickets fed on selected feeds

In this study, maturation period refers to the period within which crickets showed anatomical and morphological signs of reproductive maturation. In crickets, adult male crickets make chipping sound as a sign of the onset of reproductive maturation and have fully developed wings. Female crickets developed an ovipositor and fully developed wings and may start laying eggs. Crickets fed kales and sweet potato vines showed signs of maturity by the eighth week compared to those fed on control feed. Crickets fed on banana peels and ugali showed signs of maturity 5 weeks after the insects fed on the control feed gained maturity (14 weeks) (Figure 5).

### 3.4 Protein content

After the 14‐week period, proximate analysis was carried out to determine the protein content of the harvested crickets. Crickets fed on kales and sweet potatoes had higher (82.4% and 78.6%, respectively) proteins compared to the group fed on ugali (57.9%) and banana peels (62.9%), which is appropriate for cricket weight gain.

Although the group fed on ugali and banana peels appeared to be slightly lower in total weight gain at the end of the experiment, their protein contents (57.9 and 62.9%, respectively) were within the recommended dietary levels for human food and comparable to the popular sources of protein like fish, meat, and chicken in the Lake Victoria region. Insects fed on control feed had the lowest protein content (Table 5).

**Table 5:** Proximate protein analysis of crickets fed on kales, banana peels, sweet potato vines, ugali, and growers mash. Ash content represents the presence of minerals

Feed type	Protein content (%)	Ash content
Kales	82.4	–
Sweet potatoes	78.57	3.14
Ugali	62.88	–
Banana peels	57.87	–
Control feed	18.2	2.63

## 4 DISCUSSION

Cricket farmers have access to a variety of feed resources that included vegetables, seasonally available ingredients that are dried and milled into flour, fruit peelings, forage leaves, kitchen leftovers (mainly ugali, bread, and cooked cassava leftovers), and commercial feed. The predominantly used feeds were kales, banana peels, ugali, and sweet potato vines. Feeding has been demonstrated to be an important input in cricket production, especially in the caged crickets (Orinda et al., 2017). Both animal‐ and plant‐based feeds are important for cricket production since these insects are omnivorous (Orinda, Mosi, Ayieko, Amimo, & Nchimbi, 2018). Studies on insect nutrition have shown that variation of diet during an insect's growth, especially seasonality of its host plant, have an effect on the nutritional well‐being, oviposition, and stress tolerance ability of insects (Dunbar & Gassmann, 2013; Mabry et al., 2004; Sisodia & Singh, 2012; Van Huis et al., 2013). Some farmers have used high protein food such as chicken mash to feed cricket but these feeds are often costly (Orinda et al., 2018). Therefore, the choice of kitchen leftovers, kales, and sweet potato vines could be associated with less cost and advantage of being locally available to farmers.

Weight gained by crickets fed on kales and sweet potato vines was comparable to the growers mash as a control feed during the 14‐week experimental period. Kales and sweet potato vines may be suitable ingredients to support growth needs, especially, in the sixth to tenth week and eleventh to fourteenth week when peak biomass gain was recorded for both ingredients. This study also found that farmers could maximize weight for cricket market profitability by the use of kales substituted by sweet potato vines during the 11th‐14th weeks since this period represents the last instar. Additionally, kales are more nutritious and sweet potato vines have been shown to have a higher feeding potential in terms of quality (Baba, Nasiru, Karkarna, Muhammad, & Rano, 2018; Lefsrud, Kopsell, Wenzel, & Sheehan, 2007). The *t*‐test analysis in our study reported a statistically significant low mean weight gain for banana peels and ugali compared to cohorts fed on control feed, kales, and sweet potato vines. From the nutritional analysis of this diet, kales contained the highest amount of crude protein compared to all other feeds, while control feed (growers mash) and sweet potato vines had ash content (presence of minerals). Higher crude protein and presence of other minerals may justify high weight gain by crickets. Higher crude protein concentration in the plants might be a result of nitrogen accumulation in the young tissues which receive soluble forms of nitrogen transported from elder leaves as well (Orinda et al., 2017). Additionally, fresh kale leaves can be a good source of amino acids. Since nitrogen compounds, in which amino acids predominate, form about one‐third of the dry matter in kales (Baba et al., 2018), it is therefore expected that cricket fed on fresh vegetable leaves gain more weight compared to the other feeds.

Although this study has identified locally available feeds, several challenges emerge. For instance, kales are a staple food and sweet potato vines are predominantly used as forage leaves and their use as feed may require sustainable, chemical‐free organic production due to feed safety and food security concerns. Other concerns are that the use of sweet potato vines compromises the plants’ ability to carry out photosynthesis and develop market size tubers (Frankow‐Lindberg & Lindberg, 2003; Netsai, Moses, & Tuarira, 2019).

Generally, this study reported a maturity period of 3 months, which was similar to other studies such as Ayieko et al. (2016). However, crickets fed on kales and sweet potato vines attained maturity in a shorter period that was comparable to the control feed. Low weight recorded for crickets fed on banana peels and ugali throughout the growth period compared to kales, sweet potato vines, and the control feed indicate their suitability as feed for optimal production. Specifically, early growth stages of the nymphs require nutrients to boost growth, while 6‐ to 10‐week‐old crickets require accelerated growth and hence the feeds should be nutritious enough to support reproductive developmental needs (Lehane & Billingsley, 2012).

Studies have indicated that the use of fertilizer and soil micronutrient levels affect the chlorophyll content of maize (Janmohammadi, Navid, Segherloo, & Sabaghnia, 2016). This implies that store‐bought maize that is ground into flour to make ugali may not have the same quality. The applicability of ugali as cricket feed may further be limited by the fact that it is a staple food nationwide, thus other sustainable sources should be explored.

Proximate analysis results show that crickets fed on kales had the highest protein content followed by sweet potatoes. These findings agree with Lefsrud et al. (2007) on the nutrient content of mature kales and on the nutritional benefits of plant‐based feeds in insect diets and livestock production (Fahey, 2005; Miech et al., 2016; Oonincx, Broekhoven, Huis, & Loon, 2015; Savitha, Madalageri, Prakash, & Kumara, 2015). The findings indicated the benefits of nutritional consistency and effects of nutritional variation by maintaining either diet throughout the growth period. Additionally, kales are a potential candidate for inclusion in cricket diets to provide the required weight and nutrients, but they can be substituted by sweet potatoes. Crickets fed on sweet potato vines showed the second highest protein content, and they also attained desired weight gain required by the farmers. Although banana peels do not support desired weight required by farmers, they resulted in 57.87% protein content in crickets. This implied that they can be used in combination with other feeds to achieve desired protein content in crickets, and this can also apply to ugali as it had a similar effect.

This study has shown that different locally available feeds lead to varying weight gain and protein content in cricket. These feeds are critical input in the production process as they form part of what is transformed into body mass (Orinda et al., 2018). However, there are no specific studies on optimum feeding rates or standardized nutritional requirements and hence most studies are limited to experimental stages and cannot be applied under field conditions.

## 5 CONCLUSION

Among the popular locally available feeds, crickets performed better on kales and sweet potato vines, which indicated that they could potentially be used to replace commercial growers mash used in cricket production by local farmers. However, holistic results may be achieved if used in combination as formulated feed. Efforts to develop feed with the right nutrition for crickets should explore economically viable ingredients for small‐scale production with less competition as human food. Further research should be done to determine nutrient variation of local feed resources in different geographical zones. Additionally, more studies should be carried out on the absorption and digestibility of the ingested local feeds as well as feed conversion ratios to establish the efficiency of these feeds. Despite these findings, this study was limited to locally available feeds such as ugali, sweet potato, banana, and kales. These feeds may not be readily available in other regions and hence future studies should consider other vegetables and kitchen leftovers.

## CONFLICT OF INTEREST

The authors declare no conflict of interest.

## ETHICAL APPROVAL

This study conforms to the Declaration of Helsinki, US, and/or European Medicines Agency Guidelines for human subjects. The study protocols and procedures obtained were ethically reviewed and approved by Jaramogi Oginga Odinga University of Science and Technology Ethical Review Committee. Informed consent was obtained from all respondents and each respondent was assured of confidentiality. Animal and human testing was not necessary in this study.
